# Deciphering Pro-Lymphangiogenic Programs during Mammary Involution and Postpartum Breast Cancer

**DOI:** 10.3389/fonc.2016.00227

**Published:** 2016-11-02

**Authors:** Virginia F. Borges, Alan M. Elder, Traci R. Lyons

**Affiliations:** ^1^Young Women’s Breast Cancer Translational Program, University of Colorado Cancer Center, Aurora, CO, USA; ^2^Department of Medicine, Division of Medical Oncology, University of Colorado Anschutz Medical Campus, Aurora, CO, USA

**Keywords:** breast cancer, lymphangiogenesis, lymphatic metastasis, macrophages, postpartum

## Abstract

Postpartum breast cancers are a highly metastatic subset of young women’s breast cancers defined as breast cancers diagnosed in the postpartum period or within 5 years of last child birth. Women diagnosed with postpartum breast cancer are nearly twice as likely to develop metastasis and to die from breast cancer when compared with nulliparous women. Additionally, epidemiological studies utilizing multiple cohorts also suggest that nearly half of all breast cancers in women aged <45 qualify as postpartum cases. Understanding the biology that underlies this increased risk for metastasis and death may lead to identification of targeted interventions that will benefit the large number of young women with breast cancer who fall into this subset. Preclinical mouse models of postpartum breast cancer have revealed that breast tumor cells become more aggressive if they are present during the normal physiologic process of postpartum mammary gland involution in mice. As involution appears to be a period of lymphatic growth and remodeling, and human postpartum breast cancers have high peritumor lymphatic vessel density (LVD) and increased incidence of lymph node metastasis (1, 2), we propose that novel insight into is to be gained through the study of the biological mechanisms driving normal postpartum mammary lymphangiogenesis as well as in the microenvironment of postpartum tumors.

## Introduction to Postpartum Breast Cancer

Postpartum breast cancer is an under-recognized and highly metastatic subset of young women’s breast cancer, which we define as breast cancers diagnosed within 5 years of a women’s most recent child birth (3, 4). The distinction of postpartum cases from the various interactions of breast cancer and pregnancy, or pregnancy-associated breast cancer (PABC), arose from epidemiologic studies indicating that women diagnosed with breast cancer in the postpartum years are nearly three times as likely to develop metastasis and to die from breast cancer in comparison with nulliparous women (3–6). Importantly, this research highlighted the need to clearly separate breast cancer cases as nulliparous, pregnant, or postpartum, as opposed to defining PABC as cancers diagnosed both during and in the 1–2 years after parturition as one entity, to avoid diluting the risk signal (5). Epidemiological studies utilizing multiple cohorts also identify that ~45% of all breast cancers in Caucasian women aged ≤45 are diagnosed within 5–6 years of childbirth (5). More recently, it was identified that a breast cancer diagnosis of up to 10 years postpartum confers an ongoing measure of increased risk for metastasis, which would represent 60% or more of all young onset diagnosis in the US (7). Ideally, understanding the biology that underlies this epidemiologic risk for metastasis and death will lead to identification of targeted interventions that will benefit the large number of young women with breast cancer who fall into this subset (8).

To define the mechanisms of increased risk for metastasis, preclinical mouse models of postpartum breast cancer have revealed that tumors become more aggressive if they are exposed to the normal physiologic process of postpartum mammary gland involution. The process of postpartum breast or mammary gland involution is when the mammary epithelium regresses from the lactational state, undergoes a period of significant tissue remodeling, and resets to the pre-pregnant state. Multiple hallmarks of cancer are identified as also being important aspects of the involution process (9–37). Moreover, increased tumor growth, invasion, and metastasis are all identified when either human breast cancer or murine mammary tumors cells are implanted into postpartum hosts during involution compared with nulliparous controls (1, 36, 38–40). Mechanisms underlying this aggressive tumor promotional phenotype of involution include the induction of immunosuppression and lymphangiogenesis in the tumor microenvironment. Focusing on lymphangiogenesis, involution appears to be a period of significant lymphatic growth and remodeling, and human postpartum breast cancers have high peritumor lymphatic vessel density (LVD) and increased incidence of lymph node metastasis (2, 38, 41). Thus, we believe lymphangiogensis is an important pathway in the metastasis of postpartum breast cancer. Deeper understanding of the biological mechanisms driving normal postpartum mammary lymphangiogenesis offers potential novel insight into tumor-associated lymphangiogenesis.

## Introduction to the Lymphatic System and Lymphangiogenesis

Lymphangiogenesis is the outgrowth of new lymphatic vessels, which is required for development of the immune system, fluid homeostasis, trafficking of lymphatic cells, normal wound healing, and tissue regeneration (42–47). Differential expression of lymphatic markers, which distinguish lymphatic vessels from blood vessels, has been described in detail over the past decade and has aided the field by allowing researchers to distinguish between newly formed neo- and mature lymphatics (43, 48–57). The adult lymphatic system consists of initial lymphatics, also known as lymphatic capillaries, which drain lymph fluid into pre-collecting lymphatics, followed by drainage into collecting lymphatics that then lead to the lymph node where foreign bodies can be trapped, immune reactions occur, and lymph fluid is concentrated (42, 44). While the lymphatic endothelial marker Lyve-1 is absent in the collecting lymphatics, it is highly expressed for the initial lymphatics or lymphatic capillaries (58). Furthermore, both the lymphatic capillaries and the collecting vessels exhibit high expression levels of Prox-1, VEGFR-3, and podoplanin (48–55, 59). These results suggest that Lyve-1 may be a marker that can be used to specifically measure new lymphatic formation or neo-lymphangiogenesis.

Neo-lymphangiogenesis occurs in adult tissues as an active normal response to infection, inflammation, and wound healing. Neo-lymphangiogenesis can be stimulated by the local production of the vascular endothelial growth factors VEGF-C, and -D within the damaged tissue and subsequent binding to VEGFR-2 and VEGFR-3 on nearby lymphatic endothelial cells (LECs), resulting in the expansion of lymphatics *via* sprouting from pre-existing lymphatic vasculature (50, 51, 59–69). Primary sources of VEGF-A, -C, and -D include fibroblasts, inflammatory cells, and macrophages (70–74). An alternative theory has emerged whereby bone marrow-derived cells, specifically macrophages, may also be recruited to contribute to lymphangiogenesis (75–77). In support of this theory, bone marrow transplanted from GFP+ mice into GFP− recipients revealed GFP+ cells localized and/or incorporated into new lymphatics during inflammation, and additional lineage tracing experiments support these findings (75, 76, 78). Furthermore, tissue-resident and bone marrow-derived macrophages express lymphatic markers, such as Lyve-1, Prox-1, and podoplanin (78–80), and the presence of macrophages at sites of neo-lymphangiogenesis during inflammation has been reported (78, 80). Thus, macrophages appear to be involved in neo-lymphangiogenesis. As macrophages are also an important part of the normal program of involution (19), we believe there is a role for macrophages in facilitating the neo-lymphangiogenesis seen during postpartum mammary involution.

## Pro-Lymphatic Programs Observed During Postpartum Mammary Involution

Postpartum mammary involution has been extensively characterized using rodent mammary glands with more recent preliminary confirmation in human tissues (11, 12, 17, 19, 20, 26, 32, 35, 36, 40, 81–89). Postpartum mammary gland involution occurs in two distinct phases. During the first phase, which is reversible and lasts for 48 h (days 1–2 post weaning), apoptosis of the epithelium occurs and repopulation of the gland with adipocytes is observed. During the second phase (days 3–14), a remodeling program is initiated which results in additional cell death, increased expression of matrix remodeling proteases, degradation and remodeling of extracellular matrix components, and re-differentiation of adipocytes. Recently, we observed that neo- lymphangiogenesis occurs during postpartum mammary gland involution; however, the functional significance of these increased lymphatics has yet to be described (38, 41). Prior to our studies only a few reports had focused on mammary lymphatics, which are described below.

**Figure 1:**
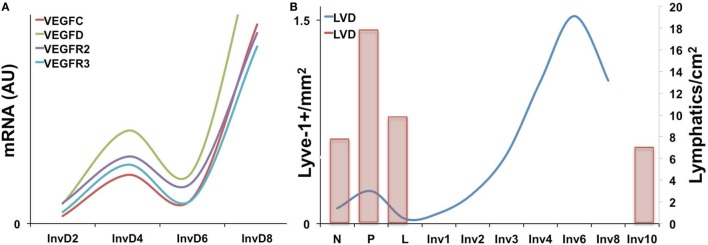
**(A)** Pro-lymphangiogenic growth factor gene expression, as measured by qPCR is increased during early and late involution in whole rat mammary tissues [adapted from Lyons et al. (38)]. **(B)** Lyve-1+ lymphatic vessels per area (left axis) is increased during pregnancy and again during involution in mouse mammary tissues with peak levels observed at day 6. (Right axis) A previous study showing a similar increase in Prox-1+ lymphatic vessels during pregnancy as well as levels at involution day 10 [adapted from Betterman et al. (91)].

Expression of the VEGF family members has been characterized during the pregnancy/lactation/involution cycle, with a goal of understanding regulation of angiogenesis in the mammary gland. In these studies, VEGF-A expression was observed as increased during pregnancy and lactation where it likely drives angiogenesis and vascular permeability, which are important for milk production. In contrast, pro-lymphangiogenic VEGFC expression levels were overall lower over the course of pregnancy and lactation, remained extremely low during the first phase of involution (days 1 and 2), and then increased nearly twofold in the second phase (days 3 and 7); this provides evidence that pro-lymphangiogenic programs may be activated during postpartum mammary involution (90). Consistent with these findings, we have observed upregulation of pro-lymphangiogenic VEGF-C and VEGF-D mRNA expression, along with their receptors, VEGFR2/3, during postpartum involution in rat mammary tissues (38) (Figure 1A). Additional studies have utilized elegant high-resolution imaging of sectioned and/or whole mounted mouse mammary glands to better understand lymphatic development during the pregnancy/lactation/involution cycle of the mammary gland. These studies revealed that VEGF-C and -D are produced locally by the mammary epithelium and myo-epithelium and that Prox-1-positive lymphatic vessels were intimately associated with the mammary epithelium and the blood vasculature.

These studies, by Betterman et al., also examined Prox-1-positive lymph vessels per area to determine density. Interestingly, they observed peak LVD during pregnancy, which decreased during lactation and involution (91). However, their analysis included only a single timepoint during involution, involution day 10, which is near the end of the involution process in mice. In contrast, while our results also reveal that the number of Lyve-1 positive vessels per area in rodent mammary glands similarly drops from pregnancy to lactation, we observe that this drop is accompanied by a subsequent rise in LVD during the early phases involution, which peaks at involution day 6 in mouse and at day 10 in rat mammary tissue (38, 41) (Figure 1B). These results suggest that neo-lymphangiogenesis occurs during the active phase of mammary remodeling in rodents. Importantly, we also analyzed podoplanin positive vessels in normal breast tissue from women who were biopsied within 10 years postpartum to determine whether the increase in lymph vessels is also evident and whether the increase persisted over time, as has been suggested by a gene signature that contains pro-angiogenic molecules angiopoietin and VEGF-A (9). The results from our study showed that women who were within 1 year of giving birth, and no longer lactating, had the highest LVD compared with never been pregnant (NBP) women. In addition, women between 3 and 10 years of giving birth also had elevated LVD compared with nulliparous suggesting that neo-lymphangiogenesis occurs during postpartum breast involution in women, and the resulting lymphatics may persist beyond the period of remodeling (38).

**Figure 2:**
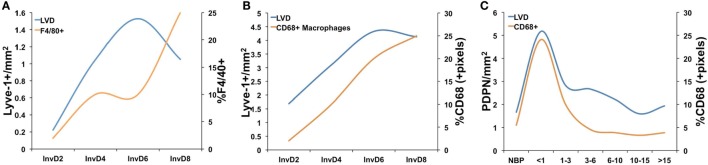
**A comparison of lymphatic vessel density (LVD) and macrophage infiltration in (A) mouse mammary tissue where macrophages were measured as %F4/80+ cells from whole mammary tissue by flow cytometry [data adapted from Lyons et al. (38) and Martinson et al. (36)], (B) rat mammary tissue where macrophages were measured by quantitative IHC as %CD68+ cells/pixel [data adapted from Lyons et al. (38) and O’Brien et al. (19)], and (C) in normal human breast tissues from women who had never been pregnant (NBP), were no longer lactating, and <1, 1–3, 3–6, 6–10, 10–15, and >15 years since last childbirth where lymphatics were measured as number of podopanin+ vessels per area and CD68 by quantitative IHC [data adapted from Lyons et al. (38) and Jindal et al. (93)]**.

Consistent with a potential role for bone marrow-derived cells in neo-lymphangiogenesis, additional studies of postpartum mammary gland involution have revealed specific changes in immune cell populations, and regulation, during the involution process (12, 19, 20, 32, 36, 40, 81–83). Initial gene expression analyses during the pregnancy/lactation/involution cycle identified upregulation of genes important for acute inflammatory responses in the mammary epithelium during postpartum involution (12, 26). Specifically, Stat3 and NF-κB are primary mediators of involution in the mouse and are also known to be key mediators of acute-phase inflammatory response. Recently, and in support of these gene expression data, the postpartum mammary gland was shown to have a cascade of infiltrating immune cells, including T-cells, T regulatory cells, and dendritic cells during involution that mimic a wound-healing pattern (92). Furthermore, numerous studies have shown that macrophages are present during involution in mouse, rat, and human tissues and that macrophage ablation during the first phase of involution in mice blocks epithelial cell death and adipocyte repopulation. The details of these studies have been reviewed elsewhere (17, 20, 81). Importantly, we observe that macrophages and lymphatics may be similarly regulated during postpartum involution in mouse, rat, and human tissues (19, 38, 93) (Figure 2). We have also shown that macrophages present during postpartum involution in rodent and human tissues express markers of an M2-polarized phenotype, such as mannose receptor, arginase-1, and CD11b (19, 36, 81). CD11b+ macrophages produce pro-lymphangiogenic factors VEGF-C and -D (78, 80, 94). Moreover, subpopulations of CD11b+ positive macrophages express lymphatic endothelial markers Lyve-1 and VEGFR-3 (95, 96). Together, these findings indicate that the CD11b+ “involution macrophages” may either stimulate lymphangiogenesis through release of pro-lymphangiogenic cytokines or through expression of lymphatic markers and incorporation into existing lymphatics; evidence for both has been published in models of inflammation and cancer (68, 78, 80, 96, 97).

Our analysis of lymphangiogenesis during postpartum involution also revealed that administration of a selective COX-2 inhibitor, celecoxib (CXB), during postpartum involution reduced Lyve-1-positive LVD at involution day 4 (38). These results are consistent with previous observations in tumor models (98–102). While, the mechanism by which CXB blocked lymphangiogenesis was not directly revealed by these studies, our *in vitro* data suggested that a product of COX-2 activity, PGE_2_, acts directly on the lymphatic endothelium *via* the EP2 receptor. However, PGE_2_ can also stimulate macrophages to an M2 phenotype (103, 104); thus, it is also possible that CXB inhibits lymphangiogenesis through macrophage-dependent mechanisms. Understanding the mechanisms underlying “involution macrophage” contribution to lymphangiogenesis during normal mammary gland development could lead to insight into macrophage-mediated lymphangiogenesis during breast cancer. We postulate that postpartum involution is a developmental window that allows for studies of mechanisms driving lymphangiogenesis.

## Lymphatic Vasculature in Breast Cancer Metastasis

While expansion of the lymphatic vasculature has been linked to faster healing and greater ability to fight infection, lymphangiogenesis can also be pathologic. Pathologic lymphangiogenesis has been observed in graft-versus-host disease, in chronic inflammatory diseases (e.g., Rheumatoid arthritis and inflammatory bowel disease), and in the tumor microenvironment. Lymph node metastasis, lymphatic vessel presence at the tumor margin, and invasion of tumor cells into peritumor lymphatics are all poor prognostic factors for breast cancer patients (105). Further, increased LVD in the peritumor region correlates with increased metastasis in a number of human cancers, directly implicating new lymphatic vessel formation in tumor cell dissemination (106–108). A multitude of studies have examined mechanisms driving neo-lymphangiogenesis in the breast tumor environment, and VEGF-C, VEGF-D, macrophages, and COX-2/PGE_2_ have emerged as key players (51, 68, 72, 73, 80, 99–101, 109–118).

VEGF-C is secreted by macrophages and other lymphatic cells to stimulate lymphangiogenesis, but can also be secreted by tumor cells for the same purpose (51, 62, 72, 73, 94, 117, 119). Macrophages have also been shown to participate directly in lymphangiogenesis *via* inducing lymphatic vessel sprouting and incorporating into existing tumor-associated lymphatics (69, 80). COX-2 and its product PGE2 also promote lymphangiogenesis in the tumor microenvironment (38, 41, 98, 99, 101, 102, 116, 120, 121). Furthermore, VEGF-D promotes lymphatic vessel dilation through a COX-2-dependent mechanism. Dilation of pre-existing, peritumor, and intratumor lymphatics allows for the intravasation of tumor cells into the lymphatic vessels and subsequent transmigration to regional lymph nodes (109, 114, 119, 122). Together these results suggest there is a connection between COX-2-mediated lymphangiogenesis and lymphogenous tumor cell spread.

## Lymphatic Vasculature in Postpartum Breast Cancer

Postpartum breast cancers are nearly three times as likely to metastasize when compared with breast cancers in nulliparous women (5, 123, 124). Furthermore, we have shown that postpartum breast cancers have increased peritumor LVD and increased lymph node involvement (1). It is anticipated that postpartum breast tumors will utilize mechanisms similar to those observed during postpartum involution to induce lymphangiogenesis. The first, and most obvious mechanism, is upregulation of COX-2 in the mammary epithelium (125), which results in increased PGE2 production to increase lymphangiogenesis. Indeed, in animals treated with celecoxib (CXB) during postpartum involution, the resultant postpartum tumors exhibit lower levels of LVD compared with untreated controls. In addition, COX-2 in the tumor cell appears to be required as well since tumors with stable siRNA knockdown of COX-2 exhibit decreased tumor-associated LVD. Finally, if postpartum tumors are re-implanted in nulliparous hosts they maintain their ability to drive tumor-associated lymphangiogenesis (38). Of interest, these results suggest that involution-induced pro-lymphangiogenic programs persist in tumor cells long after the process of involution is complete. These results are supported by gene-expression studies indicating that there is an involution signature observed in breast tissue of parous women that persists for 10 years postpartum (9).

In addition to a COX-2-dependent mechanism, if “involution macrophages” promote lymphangiogenesis during normal involution then postpartum tumor-associated macrophages (TAMs) may also acquire and utilize similar mechanisms to mediate lymphangiogenesis in the postpartum tumor microenvironment. CD11b+ is expressed by “involution macrophages” and by TAMs (96). TAMs also predict poor prognosis of breast cancer (126), and an association between TAMs and LVD has been reported for pancreatic cancer (127). Furthermore, TAMs express VEGF-C and may promote metastasis *via* lymphangiogenesis (71–73, 126, 128). Thus, the CD11b+ macrophages present during postpartum involution may promote lymphangiogenesis in a manner similar to TAMs, and we have preclinical data indicating that CD11b+ macrophages are also increased in the tumor microenvironment of involution/postpartum tumors compared with nulliparous controls (36).

## Potential Clinical Implications: Anti-Lymphangiogenic Therapy

While it is not clear why the lymphatics are expanded during postpartum involution, it is clear that postpartum tumors hijack the lymphatic vessels in the postpartum gland to drive increased metastasis. In addition, the observed tumor-promoted neo-lymphangiogenesis offers a targetable mechanism to reduce cancer metastasis (123, 124, 129–131). Anti-lymphangiogenic therapies, such as anti-VEGFR2/3 antibodies and small molecule inhibitors that target VEGFR2/3, have been tested in clinical trials for multiple solid tumor types and have shown some successes and low toxicities (132–134). Since our studies suggest that COX-2 specific inhibitors may serve to reduce tumor-associated lymphangiogenesis, we suggest that identifying whether COX-2 inhibitors can be combined with current therapies, and/or with anti-lymphangiogenesis therapy, to reduce lymphogenous spread and metastatic recurrence should be explored for postpartum breast cancer patients.

## Author Contributions

TL was responsible for overseeing writing and editing of the manuscript, assigning contributions from VB and AE, and for preparation of the figures. VB and AE contributed to writing and editing the manuscript.

## Conflict of Interest Statement

The authors declare that the research was conducted in the absence of any commercial or financial relationships that could be construed as a potential conflict of interest.
